# The Transient Receptor Potential Channel Yvc1 Deletion Recovers the Growth Defect of Calcineurin Mutant Under Endoplasmic Reticulum Stress in *Candida albicans*

**DOI:** 10.3389/fmicb.2021.752670

**Published:** 2021-11-30

**Authors:** Liping Peng, Jiawen Du, Runfan Zhang, Nali Zhu, He Zhao, Qiang Zhao, Qilin Yu, Mingchun Li

**Affiliations:** Key Laboratory of Molecular Microbiology and Technology, Ministry of Education, College of Life Sciences, Nankai University, Tianjin, China

**Keywords:** calcineurin, TRP channel, calcium transport, endoplasmic reticulum stress, *Candida albicans*

## Abstract

Transient receptor potential (TRP) channel Yvc1 was related with hyphal growth, oxidative stress response, and pathogenicity. Calcineurin subunit Cnb1 was activated immediately in yeasts when exposed to severe stimulation. However, the relationship between Yvc1 and Cnb1-governed calcium ions and endoplasmic reticulum (ER) stress response remains unrevealed. In this study, we found that the mutant *cnb1*Δ/Δ was sensitive to TN, which was related with the overexpression of membrane calcium ion channels that could increase the cytosol calcium concentration. However, the growth of the *cnb1*Δ/Δ*yvc1*Δ/Δ mutant was recovered and its cell vitality was better than the *cnb1*Δ/Δ strain. Meanwhile, the cellular calcium concentration was decreased and its fluctuation was weakened under ER stress in the *cnb1*Δ/Δ*yvc1*Δ/Δ strain. To verify the regulation role of Yvc1 in the calcium concentration, we found that the addition of CaCl_2_ led to the worse viability, while the growth state was relieved under the treatment of EGTA in the *cnb1*Δ/Δ strain. In conclusion, the deletion of *YVC1* could reduce the cellular calcium and relieve the ER stress sensitivity of the *cnb1*Δ/Δ strain. Thereby, our findings shed a novel light on the relationship between the Yvc1-governed cellular calcium concentration and ER stress response in *C. albicans*.

## Introduction

*Candida albicans*, as the most well-known pathogenic fungi, may cause lethal systemic infection of humans (Dantas Ada et al., 2015). Calcium ion is an important signal messenger, which serves various activities and structural functions in all eukaryote cells. Normally, the cytosolic calcium ions are maintained at low concentrations in *C. albicans*, in which some calcium pumps and calcium exchangers play an important role (Cagnac et al., 2010; Ghanegolmohammadi et al., 2017). However, in response to specific stress, the extracellular calcium ions enter into the cytoplasm through the high-affinity calcium uptake system (HACS) (Cyert and Philpott, 2013). HACS consists of Cch1, Mid1, and Ecm7, which interact with each other (Iida et al., 2017). Besides, the organelle vacuole is the natural calcium ion pool in yeast cells, containing over 90% of the total cellular calcium ions (Bianchi et al., 2019). Under environmental stress, the calcium ions stored in vacuolar cavity are released through transient receptor potential (TRP) channel Yvc1 into the cytosol (Palmer et al., 2001).

In *C. albicans*, calcineurin is the conserved Ca^2+^/calmodulin-dependent phosphatase, composed of catalytic subunit Cna1 and regulatory subunit Cnb1 (Connolly et al., 2018). By dephosphorylation of transcription factor Crz1, calcineurin triggers downstream signaling events and regulates the cytosol ion concentration (Chow et al., 2017; Xu et al., 2020). In *Saccharomyces cerevisiae*, calcineurin is activated when exposed to severe stimulation, such as high pH, cell membrane damage, or antifungal drugs (LaFayette et al., 2010; Li Y. et al., 2018). The most important is that calcineurin regulated *CCH1* negatively, which inhibits the expression of Cch1 once the cellular calcium ions are overloaded (Karababa et al., 2006; Teng et al., 2013; Xu et al., 2019). Yvc1 is the unique TRP-type calcium ion channel in yeast cells, a homolog with the TRP family of mammalian cells (de Castro et al., 2014). CaYvc1, similar with ScYvc1, located on the vacuolar membrane, is important for the calcium transport under environmental stress. In the previous study, we found that the deletion of *YVC1* caused hypersensitivity to oxidative stress (Yu et al., 2014b). Meanwhile, Yvc1 is important in the process of hyphal growth, and its specific localization on the vacuolar membrane is necessary for the normal function of V-ATPase in *C. albicans* (Peng et al., 2019, 2020).

Generally, the proteins are synthesized, folded, and secreted in the endoplasmic reticulum (ER) (Zhang et al., 2019). Tunicamycin (TN) inhibits N-glycosylation and blocks the formation of glycoprotein, thereby leading to ER stress (Cherepanova et al., 2019). The unfolded protein response (UPR) is the typical strategy in yeast cells for relieving the stress (Bravo et al., 2013). bZIP transcription factor Hac1, a homolog with Xbp1 in mammalian cells, is spliced at the C terminus and transported from cytosol into nucleus, triggering the immediate expression of *PMT4* or *PRB1* to alleviate the ER stress (Cheon et al., 2011; Cherry et al., 2019). In mammalian cells, the nuclear factor E2-related factor 2 (Nrf2) was activated under oxidative stress, thereby modulating ER calcium levels by the regulation of glutathione peroxidase (Granatiero et al., 2019). Besides, since ER stress contributes to intracellular calcium and stress response, overloaded calcium induced mitochondrial dysfunction in cardiac, especially complex I (Mohsin et al., 2020).

In this study, we found that *cnb1*Δ/Δ was sensitive to ER stress, which was related with the overexpression of cytoplasm membrane channel *CCH1* and irrelevant to the oxidative stress reaction. Besides, we found that the growth of the *cnb1*Δ/Δ*yvc1*Δ/Δ strain was faster than that of the *cnb1*Δ/Δ strain, and the cell death rate, vacuolar membrane permeability, and mitochondrial activity were relieved in the double mutant. Interestingly, the classical UPR pathway was activated normally in all of the strains, indicating that the mechanism of relieving the growth in *cnb1*Δ/Δ*yvc1*Δ/Δ was unrelated with the UPR pathway. However, the calcium flux was enhanced and its concentration was increased in the *cnb1*Δ/Δ strain under ER stress, and their level decreased obviously in the *cnb1*Δ/Δ*yvc1*Δ/Δ strain. CaCl_2_ or its chelating reagent EGTA was added to verify the regulatory role of Yvc1 in the calcium ion concentration, and we found that the addition of CaCl_2_ led to poor viability and weakened the functions of vacuole and mitochondria under ER stress of the *cnb1*Δ/Δ strain. However, the growth state or organelle activity was relieved under the treatment of EGTA, indicating that Yvc1 alters the cellular calcium concentration in response to ER stress to improve the *cnb1*Δ/Δ growth. Overall, our work shed a novel light on the interaction between Yvc1-mediated calcium homeostasis and ER stress response in *C. albicans*.

## Materials and Methods

### Strains and Culture Conditions

The strains and primers used in our study are listed in Tables 1, 2. Wild-type (WT) strain BWP17 was used as the background strain to construct the *cnb1*Δ/Δ, *yvc1*Δ/Δ, and *cnb1*Δ/Δ*yvc1*Δ/Δ mutant strains by the PCR-mediated homologous recombination method. The *ARG4* cassettes were amplified and transformed into the WT. The heterozygous mutants (*cnb1:ARG4*/*CNB1*) were identified by PCR. After that, the *URA3* fragment was transformed into the heterozygous mutant above to construct the *cnb1*Δ/Δ mutant strains (*cnb1:ARG4*/*cnb1:URA3*). The *yvc1*Δ/Δ and double mutant *cnb1*Δ/Δ*yvc1*Δ/Δ were constructed with a similar method.

**TABLE 1:** Strains and plasmids used in this study.

**Strains**	**Genotype and description**	**References**
*C. albicans* strains		
BWP17 (WT)	*ura3*Δ*:*λ*imm434/ura3*Δ*:*λ*imm434 his1:hisG/his1:hisG arg4:hisG/arg4:hisG*	Dana Davis
*cnb1*Δ/Δ	u*ra3*Δ*:*λ*imm434/ura3*Δ*:*λ*imm434 his1:hisG/his1:hisG arg4:hisG/arg4:hisG cnb1:ARG4/cnb1: URA3-dpl200*	This study
*yvc1*Δ/Δ	u*ra3*Δ*:*λ*imm434/ura3*Δ*:*λ*imm434 his1:hisG/his1:hisG arg4:hisG/arg4:hisG yvc1:ARG4/yvc1: URA3-dpl200*	Qilin Yu
*cnb1*Δ/Δ*yvc1*Δ/Δ	u*ra3*Δ*:*λ*imm434/ura3*Δ*:*λ*imm434 his1:hisG/his1:hisG arg4:hisG/arg4:hisG yvc1:ARG4/yvc1: URA3-dpl200 cnb1: URA3-dpl200/cnb1: URA3-dpl200*	This study
WT- pP_*CCH*__1_-GFP	*ura3*Δ*:*λ*imm434/ura3*Δ*:*λ*imm434 his1:hisG/his1:hisG arg4:hisG/arg4:hisG* pP_*CCH*__1_-GFP	This study
*cnb1*Δ/Δ-pP_*CCH*__1_-GFP	u*ra3*Δ*:*λ*imm434/ura3*Δ*:*λ*imm434 his1:hisG/his1:hisG arg4:hisG/arg4:hisG cnb1:ARG4/cnb1: URA3-dpl200* pP_*CCH*__1_-GFP	This study
*yvc1*Δ/Δ- pP_*CCH*__1_-GFP	u*ra3*Δ*:*λ*imm434/ura3*Δ*:*λ*imm434 his1:hisG/his1:hisG arg4:hisG/arg4:hisG yvc1:ARG4/yvc1: URA3-dpl200* pP_*CCH*__1_-GFP	This study
*cnb1*Δ/Δ*yvc1*Δ/Δ-pP_*CCH*__1_-GFP	u*ra3*Δ*:*λ*imm434/ura3*Δ*:*λ*imm434 his1:hisG/his1:hisG arg4:hisG/arg4:hisG yvc1:ARG4/yvc1: URA3-dpl200 cnb1: URA3-dpl200/cnb1: URA3-dpl200* pP_*CCH*__1_-GFP	This study
Plasmid		
pP_*CCH*__1_-GFP	Containing *URA3* marker, Amp^*r*^	Qilin Yu

**TABLE 2:** Primers used in this study.

**Primers**	**Sequence (5′→3′)**
ACT1-5RT	TGAGAGTTGCTCCAGAAGAAC
ACT1-3RT	GTAACACCATCACCAGAATCC
CCH1-5RT	GGAGTTGAATAATGATCCG
CCH1-3RT	TTTCCAACGACAAACATATG
PRB1-5RT	GGGGTATCTCACGTGTCAGT
PRB1-3RT	CCATTGGGCTCTATCTTCAA
PMT4-5RT	GTGGCTTCACCTTTGAAAC
PMT4-3RT	TCATCATTATGGGTCCACAT
HAC1-5RT	TGAGGATGAACACCAAGAAGAA
HAC1-3RT	TCAAAGTCCAACTGAAATGAT

Besides, the pP_*CCH*__1_-GFP plasmid was digested with *Stu*I for 1 h and transferred into the WT and other mutant strains to measure the *CCH1* expression level. In general, the strains were cultured in YPD (10 g/l yeast extract, 20 g/l peptone, 20 g/l glucose) medium. The SC (2% glucose, 0.67% yeast nitrogen base, 0.2% amino acid mixture) medium without uracil was used to separate and select the *URA3*-tagged strains.

### Spot Assay

The YPD plates containing different concentrations of TN, β-mercaptoethanol, dithiothreitol (DTT), or calcofluor white (CFW) were used to measure the sensitivity to ER stress or cell wall stress (Su et al., 2021). Besides, 5 mM reductive agent ascorbic acid (VC) was added into these stress-related plates to investigate the relationship between stress susceptibility and oxidative stress response (OSR).

### Cellular Calcium Levels

The content of cellular calcium and the calcium flux were measured with a Fluo-4 (C_51_H_50_F_2_N_2_O_23_, Beyotime, Shanghai, China, dissolved in DMSO) probe (Bartoli et al., 2019). Log-phase cells were treated with 2 μg/ml TN for 2 h. The cells were collected, washed with phosphate-buffered saline (PBS, 8 g/l NaCl, 0.2 g/l KCl, 1.42 g/l Na_2_HPO_4_, 0.27 g/l KH_2_PO_4_, pH 7.4), and resuspended with PBS buffer. The 2-mM Fluo-4 probe was added and incubated with 70 r/min at 30°C for 1 h. The fluorescence intensity (excitation wavelength at 488 nm, emission wavelength at 525 nm) was detected using a fluorescent microplate reader (Bode et al., 2020). The scanning time was sustained for 7 min with a scan gap for 1 s.

### Vacuolar Membrane Permeability Assay

5-(6)-Carboxy-2′,7′-dichlorofluorescein diacetate (C-DCFDA) or 7-amino-4 chloromethyl coumarin (CMAC) was used to measure the vacuolar membrane permeability (VMP) (Andrei-Selmer et al., 2001). In the normal cells, C-DCFDA with green fluorescence or CMAC with blue fluorescence was concentrated in the vacuolar cavity, while it was spread to the whole cell in VMP-positive cells. The TN-treated strains were resuspended in PBS buffer and added with 1 mg/ml C-DCFDA (C_25_H_14_Cl_2_O_9_, Heowns, Tianjin, China, dissolved in DMSO) or 1 mg/ml CMAC (C_10_H_8_ClNO_2_, Beyotime, China, dissolved in DMSO). The cells were incubated for 10 min and observed by fluorescence microscopy. At least 1,000 cells for each group were photographed to count the percent of VMP-positive cells.

### Mitochondrial Membrane Potential (ΔΨm) Assays

The log-phase cells treated with 2 μg/ml TN were collected, washed, and resuspended in PBS buffer. Two micrograms per milliliter of JC-1 (2 mg/ml, dissolved in DMSO, Sigma) was added into the suspension. The cells were incubated for 1 h, and the mitochondrial membrane potential (MMP) was recorded with the flow cytometer (FACSCalibur, BD, San Jose, CA, United States). The red fluorescence (excitation wavelength at 525 nm, emission wavelength at 590 nm) with J-aggregates could be detected in the cells with normal MMP. The green fluorescence (excitation wavelength at 490 nm, emission wavelength at 530 nm) with J-monomer was detected in the cells with decreased MMP (Yu et al., 2021).

### *HAC1* Splicing Assay

The strains treated with TN were cultured to log-phage. Total RNA was extracted with Eastep^TM^ Total RNA Extraction Kit (Promega, Madison, WI, United States) and transcribed reversely to cDNA. The *HAC1* splicing assay was detected by the PCR method with primers HAC1-5RT and HAC1-3RT. The PCR product was separated in agarose gel electrophoresis for 3 h (Li J. et al., 2018).

### MTT Assay

The cellular viability was detected by MTT reagent [3-(4,5)-dimethylthiazo(-z-y1)-3,5-di-phenytetrazoliumromide, 4 mg/ml, Beyotime, China] (Rong et al., 2020). The log-phage cells were collected and resuspended in PBS buffer. The 0.5-mg/ml MTT reagent was added and incubated with 70 r/min at 37°C for 1 h. The cells were centrifuged, and the supernatant was removed. Dimethyl sulfoxide (DMSO, Beyotime, China) was added into the precipitated cells, and the cells were centrifuged. The dissolved supernatant was collected to measure the absorption wavelength at 570 nm.

### Propidium Iodide Assay

Propidium iodide (PI) was used to measure the cell death rate for the PI-positive cells which were stained with red fluorescence completely (Priante et al., 2018). The strains treated with TN were cultured to log-phase. Five micrograms per milliliter of PI (1 mg/ml, Sigma) was added into cells and incubated for 5 min. Mortality rate was represented by the PI-positive cells with a flow cytometer.

### Real-Time PCR Assay

Real-time PCR assay was used to measure the expression level of the calcium channel-related gene *CCH1* and UPR response-related genes *PMT4* and *PRB1*. Cells were collected, and the total RNA was extracted with Eastep^TM^ Total RNA Extraction Kit (Promega, United States) and transcribed reversely to cDNA (Meng et al., 2018). The RealMasterMix (SYBR Green) kit (TransGen, China) was used for real-time PCR analysis (Gomes-Neto et al., 2017). The following primers used are listed in Table 2: ACT1-5RT, ACT1-3RT, CCH1-5RT, CCH1-3RT, PRB1-5RT, PRB1-3RT, PMT4-5RT, and PMT4-3RT. The 2^–ΔΔ^ CT method was used to calculate the expression level of different genes, and *ACT1* was used as the internal control.

### Statistical Analysis

Each experiment mentioned above was repeated at least three times under the tested conditions. The standard deviations and means were calculated by the separate three replicates. The one-tailed Student’s *t* test was used to calculate *p* values. The *p* values less than 0.05 were considered as statistically significant difference.

## Results

### The Deletion of *CNB1* Caused Sensitivity to Endoplasmic Reticulum Stress and Led to the *CCH1* Overexpression

**FIGURE 1:**
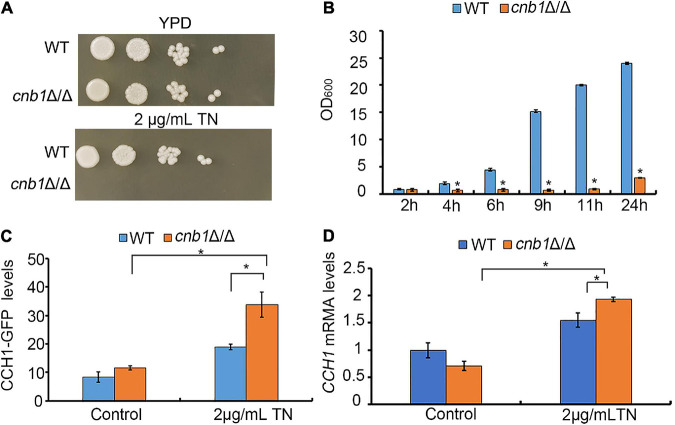
Effect of the *CNB1* deletion on sensitivity to ER stress. **(A)** Cells were spotted on the YPD plates containing TN and photographed after being cultured for 2–3 days. **(B)** The liquid growth assay of *cnb1*Δ/Δ. **(C)** The CCH1-GFP fluorescence levels under ER stress. Strains tagged with the CCH1-GFP fragment were cultured to log phase, and the fluorescence intensity was detected with fluorescent microplate reader. **(D)** The *CCH1* expression level. Total RNA was extracted and transcribed reversely to cDNA. CCH1-5RT CCH1-3RT were used for real-time quantitative PCR analysis. * means significant difference between the mutant and WT strains (*p* < 0.05). The experiments above were repeated three times independently.

Firstly, we constructed the *cnb1*Δ/Δ mutant and measured its sensitivity to ER stress reagent TN. The results showed that on the YPD plate, *cnb1*Δ/Δ grew normally as a wild-type (WT) strain, while *cnb1*Δ/Δ could hardly grow on the 2-μg/ml TN plate (Figure 1A). Besides, the growth of liquid medium indicated that the WT strain grew rapidly during the 24-h culture period, whose value increased from 0.8 to 24. However, the OD_600_ of *cnb1*Δ/Δ was always maintained at 0.8 and the maximum value at 24 h was just 3 (Figure 1B). Since calcineurin regulated the cellular calcium concentration through inhibition of plasma membrane channel *CCH1* (Xu et al., 2019), we assumed that the deletion of *CNB1* might have an impact on the *CCH1* expression. It was interesting that under TN treatment, the expression level of the *CCH1* promoter in the *cnb1*Δ/Δ strain was near as 1.5 times as WT (Figure 1C). Moreover, the qPCR analysis indicated that *CCH1* was upregulated in the transcription level of *cnb1*Δ/Δ in response to ER stress (Figure 1D). In general, the deletion of *CNB1* leads to the sensitivity to ER stress and overexpression of *CCH1*.

### The Inhibition of the Plasma Membrane Calcium Channel Could Recover the Growth Defect of *cnb1*Δ/Δ Under Endoplasmic Reticulum Stress

**FIGURE 2:**
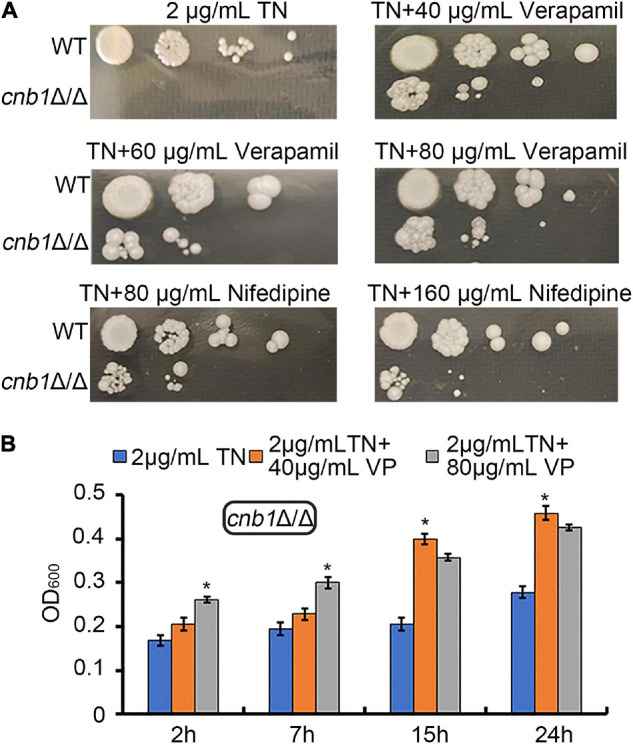
The supplement of verapamil and nifedipine recovered the growth defect of *cnb1*Δ/Δ under ER stress. **(A)** The different concentrations of verapamil and nifedipine were added into TN-containing YPD plates. WT and *cnb1*Δ/Δ strains were cultured and spotted on these plates. **(B)** Forty micrograms per milliliter of verapamil or 80 μg/ml verapamil was added to the TN-treated YPD medium. Strains were cultured, and OD_600_ was measured at 2, 7, 15, and 24 h. * means significant difference between TN treated alone and TN with verapamil-treated strains (<0.05). The experiments above were repeated three times independently.

Now that the sensitivity to ER stress of the *cnb1*Δ/Δ strain was related with the overexpression of *CCH1*, we speculated that the inhibition of the plasma membrane calcium channel might improve its growth status. Verapamil or nifedipine, as the universal calcium channel blocker, was usually applied to cure the hypertension or angina. A different concentration of verapamil or nifedipine was added into the YPD plates containing 2 μg/ml TN. The spot assay result showed that the colony of *cnb1*Δ/Δ could grow in both the plates treated with verapamil and nifedipine (Figure 2A). Meanwhile, the liquid growth measurement indicated that during the 24-h culture, 40 or 80 μg/ml verapamil could improve the growth of *cnb1*Δ/Δ under ER stress (Figure 2B). These results implied that the inhibition of the plasma membrane calcium channel to decrease the cytosolic calcium concentration could recover the *cnb1*Δ/Δ growth under ER stress.

### Deletion of *YVC1* Decreased the Cell Death Rate of *cnb1*Δ/Δ Under Endoplasmic Reticulum Stress

**FIGURE 3:**
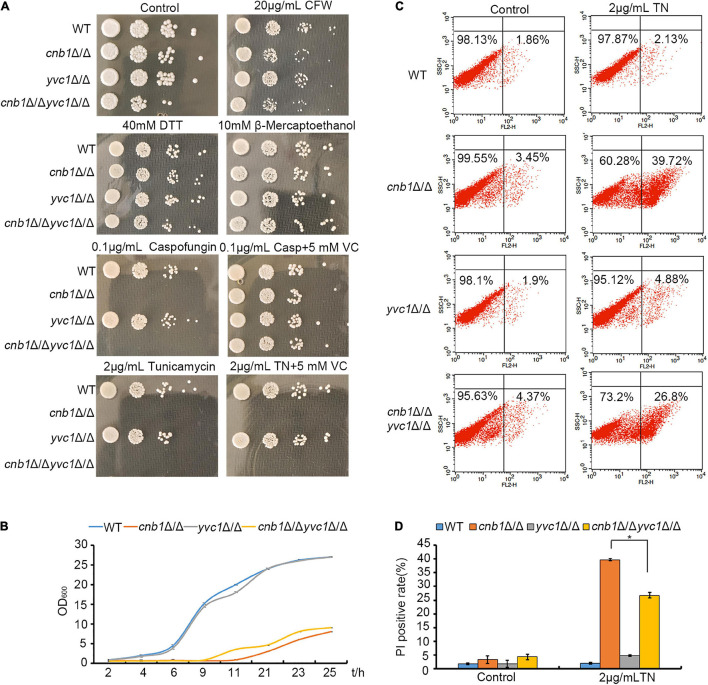
Deletion of *CNB1* and *YVC1* recovered the growth defect of the *cnb1*Δ/Δ strain. **(A)** ER stress or cell wall stress-related reagents were added into YPD plates. WT, *cnb1*Δ/Δ, *yvc1*Δ/Δ, or *cnb1*Δ/Δ*yvc1*Δ/Δ strains were cultured and spotted on the plates. **(B)** The growth curve of each strain was drawn. The initial OD_600_ value was rectified at 0.2 and strains were cultured in liquid YPD medium, and 2 μg/ml TN was added into these medium and cultured for 25 h. The OD_600_ value in each strain was measured at specific times. **(C)** The death rate of each strain under ER stress. Log-phase strains were collected and washed twice with PBS buffer. Five micrograms per milliliter of PI was added into the strains and incubated for 5 min. The cell death rate was measured by flow cytometry. The cells without TN treatment were as the control group. **(D)** The statistical analysis of PI death rate. * means significant difference between the *cnb1*Δ/Δ and *cnb1*Δ/Δ*yvc1*Δ/Δ strains under TN-treated conditions (*p* < 0.05). The experiments were repeated three times separately.

Since the inhibition of the cytosolic calcium concentration recovered the growth of *cnb1*Δ/Δ and Yvc1 was the unique vacuolar membrane calcium channel in yeast, we doubted whether the deletion of *YVC1* could improve the growth status of *cnb1*Δ/Δ under ER stress. We constructed *yvc1*Δ/Δ and *cnb1*Δ/Δ*yvc1*Δ/Δ strains and measured their susceptibility to environmental stress. CFW and caspofungin could cause the cell wall stress. Besides, both β-mercaptoethanol and DTT could interfere the formation of disulfide bonds, and TN could inhibit glycosylation. These three reagents could lead to ER stress. We found that similar with the WT strain, *cnb1*Δ/Δ, *yvc1*Δ/Δ, and *cnb1*Δ/Δ*yvc1*Δ/Δ strains were resistant to CFW, β-mercaptoethanol, and DTT (Figure 3A, panels 2–4). However, the *cnb1*Δ/Δ and *cnb1*Δ/Δ*yvc1*Δ/Δ strains were sensitive to caspofungin and TN. Since Yvc1 regulated the OSR in yeast (Yu et al., 2014b), the antioxidant vitamin C (VC) was added into the plates to verify whether the sensitivity was related with abnormal OSR. It was interesting that the addition of VC recovered the strains on the caspofungin-treated plate, indicating that the sensitivity to cell wall stress was related with impaired OSR reaction (Figure 3A, panels 5–6). Nevertheless, the addition of VC could not change the strains’ susceptibility to TN (Figure 3A, panels 7–8), which revealed the novel interaction between Yvc1 and Cnb1 with ER stress independently of OSR.

Under TN treatment, we measured the strain growth in the liquid medium during the 24-h cultivation. WT and *yvc1*Δ/Δ strains grew rapidly to the log phase and eventually maintained with the maximum quantity. Although *cnb1*Δ/Δ and *cnb1*Δ/Δ*yvc1*Δ/Δ mutants grew slowly, *cnb1*Δ/Δ*yvc1*Δ/Δ grew marginally faster than the *cnb1*Δ/Δ strain did (Figure 3B). Besides, the cell death rates under ER stress of these strains were calculated by flow cytometry with PI dye. All of the strains grew well in the control group, and their dead rates were low (Figures 3C,D, control). Under the TN treatment, WT and *yvc1*Δ/Δ strains were still with the small dead cells, and the death rate was 26.8% in the *cnb1*Δ/Δ*yvc1*Δ/Δ strains, which was increased to 39.72% in the *cnb1*Δ/Δ strain (Figures 3C,D). These results indicated that even if *cnb1*Δ/Δ*yvc1*Δ/Δ showed sensitivity to TN, the cell death rate was lower than that of the *cnb1*Δ/Δ strain, indicating that the deletion of *YVC1* recovered the cell vitality of *cnb1*Δ/Δ.

### The Deletion of *YVC1* Recovered Vacuolar Membrane Permeability and Mitochondrial Membrane Potential of *cnb1*Δ/Δ Under Endoplasmic Reticulum Stress

**FIGURE 4:**
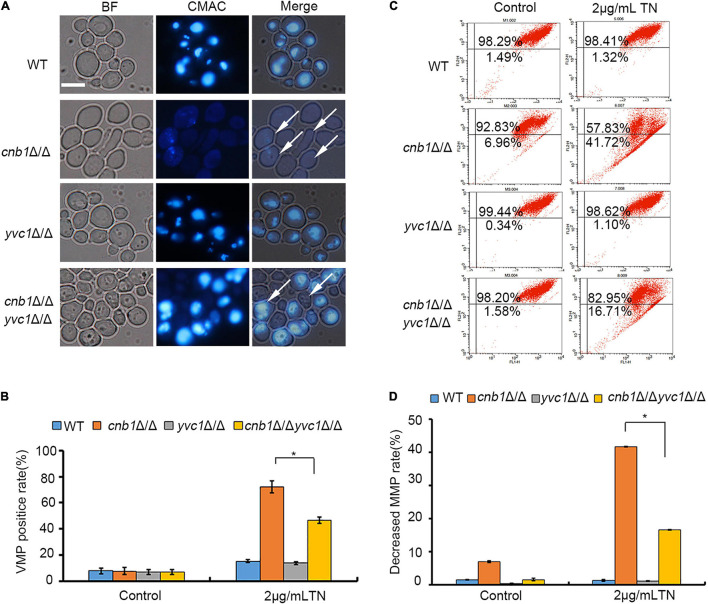
The deletion of *YVC1* recovered the vacuolar membrane permeability (VMP) and mitochondrial membrane potential (MMP) under ER stress. **(A)** The detection reagent CMAC was added into the TN-treated strains. The cells were incubated with a gentle shaker at 30°C for 30 min and observed by fluorescence microscopy for each group. White arrows indicated the VMP-positive cells. Bar = 10 μm. **(B)** The calculation of VMP-positive cells. At least 1,000 cells in each strain were photographed and counted. **(C)** The log-phase cells were collected and resuspended in PBS buffer. JC-1 was added into the cells and incubated. The mitochondrial membrane potential (MMP) was detected by flow cytometry. **(D)** The calculation of MMP-decreased cells. The experiments were repeated three times separately. * means significant difference between the *cnb1*Δ/Δ and *cnb1*Δ/Δ*yvc1*Δ/Δ strains under TN-treated conditions (*p* < 0.05).

Normally, the vacuole cavity could be dyed by CMAC, while the damaged cells were stained in the whole cell or failed to be stained (Andrei-Selmer et al., 2001). The vacuolar membrane permeability of the mutants showed that deletion of *CNB1* caused the damaged vacuolar membrane under TN treatment, with the whole cells stained by CMAC or failing to be stained (Figure 4A). Besides, the calculation of the VMP-positive rate showed that cultured in the TN-treated medium, the WT and *yvc1*Δ/Δ strains maintained the integrity of vacuoles, with a low percentage of the VMP-positive rate. However, the positive rate was up to 70% in the vacuolar severely impaired *cnb1*Δ/Δ strain, although for the *cnb1*Δ/Δ*yvc1*Δ/Δ strain with impaired vacuolar membrane, the VMP-positive rate was lower than *cnb1*Δ/Δ, just about 45% (Figure 4B).

The mitochondrial activities under ER stress were measured as well. In the control group, the *cnb1*Δ/Δ*yvc1*Δ/Δ, *cnb1*Δ/Δ, and *yvc1*Δ/Δ strain, as the WT strain, maintained the normal mitochondrial function, with the low percentage of damaged rate (Figures 4C,D, control). However, compared with the other strains, the mitochondrial function was interfered by TN in the *cnb1*Δ/Δ strain, and the rate of decreased MMP was up to 41.72%. Nevertheless, the rate of impaired mitochondria of *cnb1*Δ/Δ*yvc1*Δ/Δ was just 16.71% (Figures 4C,D). In conclusion, the deletion of *YVC1* recovered the function of vacuole and mitochondria of the *cnb1*Δ/Δ strain.

### The Unfolded Protein Response Pathway Was Activated Effectively in the *cnb1*Δ/Δ and *cnb1*Δ/Δ*yvc1*Δ/Δ Strains Under Endoplasmic Reticulum Stress

**FIGURE 5:**
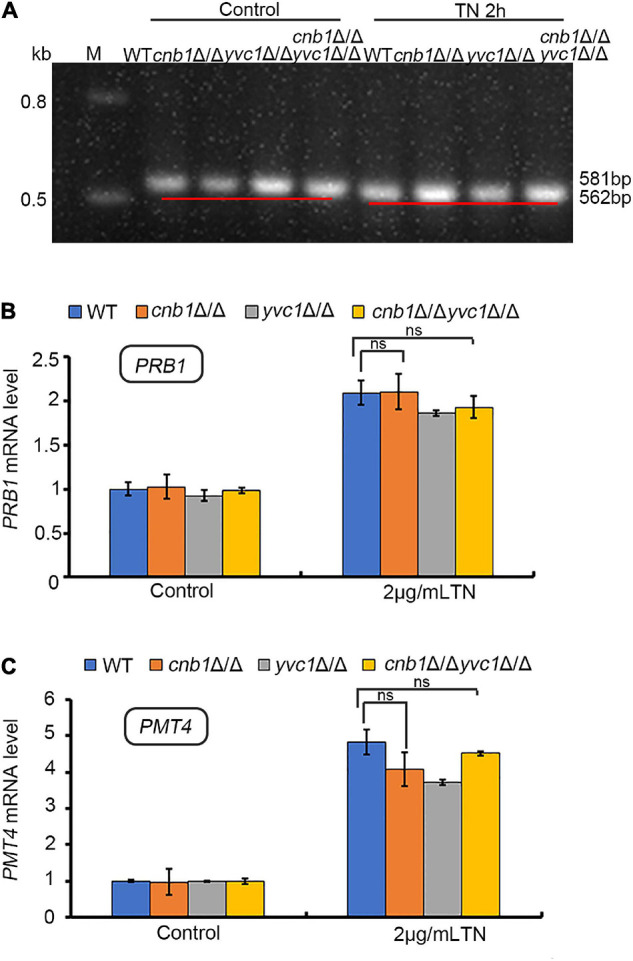
UPR pathway was activated effectively in both WT and other mutant strains under ER stress. **(A)** HAC1 splicing assay. The primers HAC1-5RT and HAC1-3RT were used to amplify the *HAC1* fragment. **(B)** UPR gene *PRB1* expression levels. The primers PRB1-5RT and PRB1-3RT were used to amplify the cDNA fragment of *PRB1*. **(C)** Real-time quantitative PCR to detect the expression level of *PMT4*. “ns” means no significant difference between the WT and other mutant strains under TN-treated conditions (*p* < 0.05). The experiments above were repeated three times independently.

The UPR pathway is the classical response process in the ER stress of yeast strains (Zhang et al., 2019). Since the deletion of *YVC1* recovered the cell vitality of *cnb1*Δ/Δ under the treatment of TN, we speculated whether the UPR pathway was overactivated in this mutant. To verify the possibility, the total RNA of these three mutants and WT strains was extracted and transcribed reversely to cDNA (Gomes-Neto et al., 2017). The Hac1 splicing level and the expression level of *PRB1* and *PMT4* were measured. However, much unexpectedly, similar with the WT or *yvc1*Δ/Δ mutants, the UPR pathway was activated in *cnb1*Δ/Δ and *cnb1*Δ/Δ*yvc1*Δ/Δ effectively. The unspliced *HAC1* was 581 bp in the WT and mutant strains of the control group (Figure 5A). Moreover, under TN treatment for 2 h, *HAC1* was spliced normally in the *cnb1*Δ/Δ and *cnb1*Δ/Δ*yvc1*Δ/Δ strains and other strains with the size at 562 bp (Figure 5A).

Besides, qPCR analysis indicated that the expression levels of *PMT4* and *PRB1* in the *cnb1*Δ/Δ and *cnb1*Δ/Δ*yvc1*Δ/Δ strains were similar with WT, which were all upregulated in the transcriptional level under ER stress (Figures 5B,C). In summary, these figures showed that the URP pathway in all of the mutants did not fail to evoke, implying a novel regulation mechanism within susceptibility.

### The Deletion of *YVC1* Decreased the Calcium Fluctuation and Cellular Calcium Concentration of *cnb1*Δ/Δ Under Endoplasmic Reticulum Stress

**FIGURE 6:**
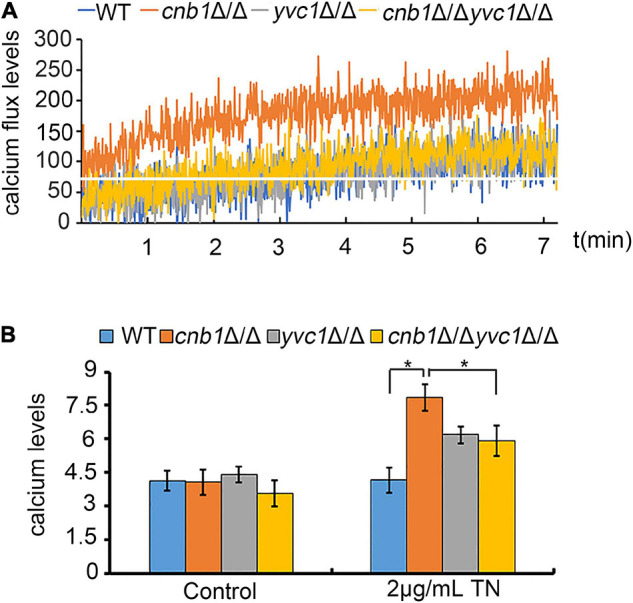
Deletion of *CNB1* and *YVC1* caused decreased calcium flux and calcium concentration. **(A)** The calcium flux assay. The experiment was detected for 7 min with a 1-s gap by a fluorescent microplate reader. The white straight line indicated the initial calcium concentration of *cnb1*Δ/Δ. **(B)** The cellular calcium concentration was detected with the fluorescent value in which the excitation wavelength was 488 nm and the emission wavelength was 525 nm. * means significant difference between the WT and other mutant strains under TN-treated conditions (*p* < 0.05).

Yvc1 and Cnb1 were related with the cellular calcium regulation (Cyert and Philpott, 2013), and the inhibition of *CCH1* to decrease the cytosolic calcium content could improve the growth status in the *cnb1*Δ/Δ strain. Therefore, we speculated whether the disruption of *YVC1* reduces the vacuolar calcium release to improve the cell vitality. Moreover, the results showed that calcium fluctuation was enhanced in the *cnb1*Δ/Δ strain under ER stress. In the detected period, the calcium fluctuation was increased within 3 min, and the maximum concentration was higher than other strains. Nevertheless, the calcium flux of the *cnb1*Δ/Δ*yvc1*Δ/Δ strain was steady with a low peak value like the WT strain (Figure 6A).

Next, we measured the specific concentration of cellular calcium, which showed a similar tendency with the calcium flux. We found that the calcium concentration was increased significantly with TN treatment for 2 h of *cnb1*Δ/Δ, in which the concentration was much higher than those of other strains. The calcium content of the *cnb1*Δ/Δ*yvc1*Δ/Δ strain was close to that of *yvc1*Δ/Δ, lower than that of *cnb1*Δ/Δ (Figure 6B). In conclusion, the calcium concentration was decreased significantly and its fluctuation was reduced obviously under ER stress in the *cnb1*Δ/Δ*yvc1*Δ/Δ strain, indicating that the disruption of *YVC1* could reduce the cellular calcium concentration, thereby improving the vitality.

### The Supplement of EGTA Could Recover the Growth of *cnb1*Δ/Δ Under Endoplasmic Reticulum Stress

**FIGURE 7:**
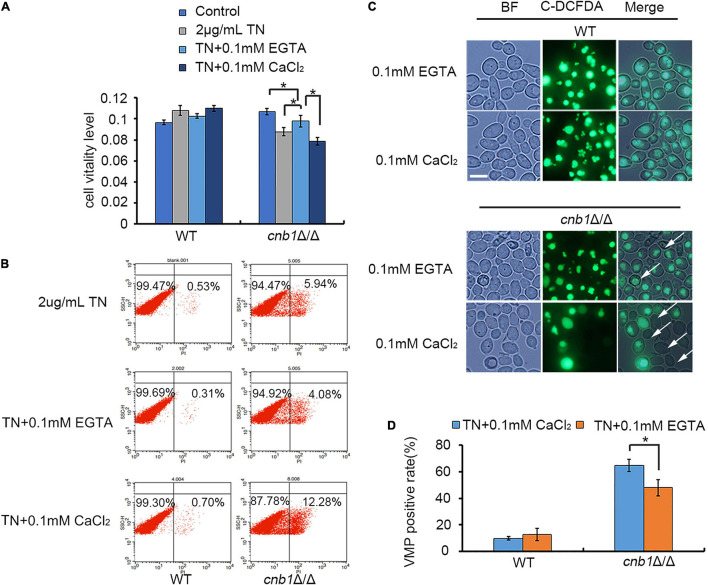
The supplement of EGTA could recover the cell viability of the *cnb1*Δ/Δ strain under ER stress. **(A)** WT and *cnb1*Δ/Δ were cultured in the medium containing TN supplied with EGTA or CaCl_2_. The MTT reagent was added, and the absorption wavelength of 570 nm was detected. **(B)** After the supplement of EGTA or CaCl_2_ into the TN-treated *cnb1*Δ/Δ, the PI death rate was measured by flow cytometry. **(C)** The observation of vacuolar membrane permeability under TN treatment supplied with EGTA or CaCl_2_. The method was similar with that in Figure 4A. Bar = 10 μm. **(D)** The count of VMP-positive cells treated by TN supplied with CaCl_2_ or EGTA. The method was similar with that in Figure 4B. * means significant difference among the different treated strains (*p* < 0.05). The experiments were repeated three times separately.

**FIGURE 8:**
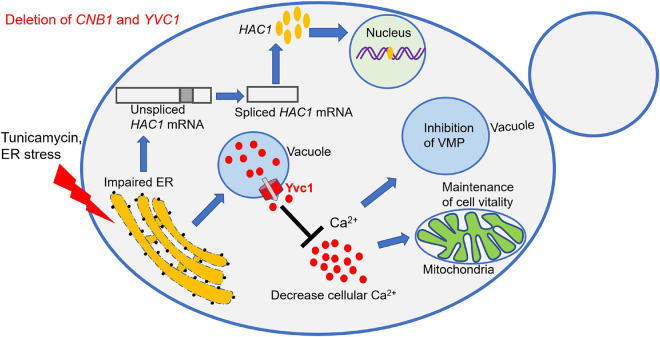
Schematic model of Yvc1 regulation cellular calcium concentration to improve growth in the *cnb1*Δ/Δ*yvc1*Δ/Δ strain under ER stress. The calcineurin mutant strain *cnb1*Δ/Δ was sensitive to ER stress while the double mutant *cnb1*Δ/Δ*yvc1*Δ/Δ recovered the growth. The URP pathway was effectively activated in these mutants. However, the deletion of TRP channel Yvc1 could inhibit the release of Ca^2+^ from vacuole and reduce the cellular calcium concentration in response to ER stress. Thereby, the vacuolar membrane permeability (VMP) was inhibited and the cell vitality was maintained, leading to the improvement of cell growth.

To test and verify our hypothesis that the deletion of *YVC1* could decrease the cytosolic calcium concentration and recover the cell growth, CaCl_2_ or its chelating agent EGTA was added into the culture medium in the presence of TN. Although TN treatment in *cnb1*Δ/Δ caused a decreased MTT level, the supplement of EGTA recovered the cellular vitality, in which the MTT level was increased. Moreover, the supplement of CaCl_2_ led to significantly decreased vitality level in *cnb1*Δ/Δ (Figure 7A). Moreover, the PI death rate in the EGTA group was 4.08%, which increased to 12.28% in the CaCl_2_ group. It indicated that the addition of EGTA improved the cell growth as well (Figure 7B). The VMP assay showed that the permeability was improved in the addition of EGTA, while CaCl_2_ caused severely damaged VMP in the *cnb1*Δ/Δ strain (Figure 7C). Furthermore, the VMP-positive rate calculation displayed that under TN treatment, the addition of CaCl_2_ led to a 60% positive rate of the mutant, while under EGTA treatment, the positive rate was down to 48% (Figure 7D). In conclusion, we determine that it is the overloaded calcium ions that cause the susceptibility to ER stress in the *cnb1*Δ/Δ strain, and the disruption of *YVC1* reduces the cytosolic calcium and improves the cell vitality (Figure 8).

## Discussion

In this study, we found that the deletion of *YVC1* recovered the growth defect of *cnb1*Δ/Δ in the regulation of calcium ions in response to ER stress (Figure 8). *cnb1*Δ/Δ was hypersensitive to TN, which was relevant with the overexpression of the cytoplasm membrane channel *CCH1* (Figures 1, 2). This revealed the interaction between the sensitivity to ER stress and the regulation of cytosolic calcium content of the *cnb1*Δ/Δ strain. Although *cnb1*Δ/Δ*yvc1*Δ/Δ was sensitive to TN, the growth was recovered and the death rate was obviously decreased (Figure 3). In the spot assay, the strains were sensitive to TN instead of DTT, the former inhibited the N-glycosylation, and the latter influenced the disulfide bond. The results indicated that the disruption strain might have no impact on the disulfide bond, which could be verified subsequently. Moreover, the vacuolar membrane permeability and mitochondrial activity were improved in the double mutant (Figure 4).

The UPR pathway was a classical regulation method in response to ER stress; much unexpectedly, the UPR pathway was evoked in all of the tested strains, indicating their normal activation (Figure 5). However, we measured the calcium flux and the cellular calcium content. The calcium fluctuation was enhanced with the highest ion concentration in the *cnb1*Δ/Δ strain. Also, the calcium flux was steady with a lower content in the *cnb1*Δ/Δ*yvc1*Δ/Δ strain, indicating the effect of the deletion of *YVC1* on the decrease in cellular calcium (Figure 6). The further results show that addition of EGTA recovered the cell growth, cell vitality, and vacuolar membrane permeability, which corresponded with our conjectures (Figure 7).

Endoplasmic reticulum stress leads to the activation of the IRE1/Xbp1 signal pathway in mammalian cells. Regulated by Hac1, Pmt4 is expressed in the nucleus to alleviate the misfolded or unfolded proteins. In *S. cerevisiae*, ER stress upregulated the expression of Ptp2 tyrosine phosphatase and Cmp2 calcineurin phosphatase; the former was mediated by Mpk1 MAP kinase, and the latter could downregulate the activity of Hog1 MAP kinase (Mizuno et al., 2018). The Cmp2 homolog with Cna1 in *C. albicans* is the catalytic subunit which maintains the principal role for the stress response. Besides, calcineurin is essential for cells in many biological processes and for the long-term survival of cells undergoing ER stress (Liu et al., 2015). Connected with our findings, we doubt whether the Hog1-MAPK pathway is related with the ER stress response in the mutants.

It was reported that for humans, the nephrotic syndrome was associated with the activation of the ER calcium release channel, which led to podocyte injury (Park et al., 2019). Moreover, the connection of ER and mitochondria was complex. Through shuttling of calcium ions, the connection was involved not only in ion homeostasis but also in many structural and apoptotic proteins (Kumar and Maity, 2021). A stimulator of IFN genes, called STING, regulated not only calcium homeostasis but also ER stress and T cell survival, which were associated with lung disease (Wu et al., 2019). In yeast, cadmium exposure led to the interrupted calcium homeostasis, induced the lipid dysregulation, and finally caused ER stress (Rajakumar et al., 2016).

Verapamil and nifedipine are the cytoplasm membrane channel blockers in mammalian cells, used as the clinical drugs in curing heart diseases like angina pectoris and supraventricular arrhythmias (Xing et al., 2020). In our previous work, verapamil inhibited the Hwp1 expression, indicating its regulatory role in both morphogenesis-associated proteins and the secretory pathway (Yu et al., 2014a). Moreover, the combined use of verapamil and antifungal drug fluconazole had a synergetic inhibitory effect on hyphal development (unpublished data). Thereby, the cumulative effect of verapamil and TN could be measured in-depth. Besides, the effect of *CNB1* or *YVC1* deletion was interesting since the sensitivity to TN was irrelevant with the UPR pathway; indeed, it was the ROS-independent and UPR-independent calcium overloading. Connected with our previous cognition, it was subversive and enlightening.

Moreover, the relationship between the calcium signaling pathway members and the environmental stress response needs further investigation, for instance, the relationship between Cch1 and Yvc1 for their regulation role in calcium ion content. Since Cnb1 regulates the ER stress response, does the transcription factor Crz1 or the other subunit of calcineurin Cna1 regulate *CCH1* in *C. albicans*. Besides, the specific regulation site of *CCH1*, the mechanism by which deletion of Cnb1 and Yvc1 caused cellular calcium ions to be increased under TN treatment, and the sensitivity to other environmental stimulus of the mutant are still unknown. Furthermore, we will work in-depth to figure out more mechanisms about the calcium signal pathway regulation under environmental stress.

In summary, our study revealed a novel interaction between the Yvc1-regulated cellular calcium ions and ER stress response, which was independent on the antioxidative reaction or UPR pathway (Figure 8). This work will extend our knowledge of the cellular sensor role of the TRP channel and calcineurin under environmental stress and uncover the new targets against fungal infections.

## Data Availability Statement

The original contributions presented in the study are included in the article/supplementary material, further inquiries can be directed to the corresponding author/s.

## Author Contributions

LP and ML conceived and designed the experiments and wrote the manuscript. LP, JD, and RZ performed the experiments. HZ, NZ, and QZ analyzed the data. ML and QY did supervision. All authors have read and agreed to the published version of the manuscript.

## Conflict of Interest

The authors declare that the research was conducted in the absence of any commercial or financial relationships that could be construed as a potential conflict of interest.

## Publisher’s Note

All claims expressed in this article are solely those of the authors and do not necessarily represent those of their affiliated organizations, or those of the publisher, the editors and the reviewers. Any product that may be evaluated in this article, or claim that may be made by its manufacturer, is not guaranteed or endorsed by the publisher.

## References

[B1] Andrei-Selmer C. Knuppel A. Satyanarayana C. Heese C. Schu P. V. (2001). A new class of mutants deficient in dodecamerization of aminopeptidase 1 and vacuolar transport. *J. Biol. Chem.* 276 11606–11614. 10.1074/jbc.M003846200 11152450

[B2] Bartoli F. Moradi Bachiller S. Antigny F. Bedouet K. Gerbaud P. Sabourin J. (2019). Specific upregulation of TRPC1 and TRPC5 channels by mineralocorticoid pathway in adult rat ventricular cardiomyocytes. *Cells* 9:47. 10.3390/cells9010047 31878108 PMC7017140

[B3] Bianchi F. Van’t Klooster J. S. Ruiz S. J. Poolman B. (2019). Regulation of amino acid transport in *Saccharomyces Cerevisiae*. *Microbiol. Mol. Biol. Rev.* 83 e00024–e00119. 10.1128/mmbr.00024-19 31619504 PMC7405077

[B4] Bode D. Wen Y. Hegemann N. Primessnig U. Parwani A. Boldt L. H. (2020). Oxidative stress and inflammatory modulation of Ca(2+) handling in metabolic HFpEF-related left atrial cardiomyopathy. *Antioxidants (Basel)* 9:860. 10.3390/antiox9090860 32937823 PMC7555173

[B5] Bravo R. Parra V. Gatica D. Rodriguez A. E. Torrealba N. Paredes F. (2013). Endoplasmic reticulum and the unfolded protein response: dynamics and metabolic integration. *Int. Rev. Cell Mol. Biol.* 301 215–290. 10.1016/b978-0-12-407704-1.00005-1 23317820 PMC3666557

[B6] Cagnac O. Aranda-Sicilia M. N. Leterrier M. Rodriguez-Rosales M. P. Venema K. (2010). Vacuolar cation/H+ antiporters of *Saccharomyces cerevisiae*. *J. Biol. Chem.* 285 33914–33922. 10.1074/jbc.M110.116590 20709757 PMC2962491

[B7] Cheon S. A. Jung K. W. Chen Y. L. Heitman J. Bahn Y. S. Kang H. A. (2011). Unique evolution of the UPR pathway with a novel bZIP transcription factor, Hxl1, for controlling pathogenicity of *Cryptococcus neoformans*. *PLoS Pathog.* 7:e1002177. 10.1371/journal.ppat.1002177 21852949 PMC3154848

[B8] Cherepanova N. A. Venev S. V. Leszyk J. D. Shaffer S. A. Gilmore R. (2019). Quantitative glycoproteomics reveals new classes of STT3A-and STT3B-dependent N-glycosylation sites. *J. Cell Biol.* 218 2782–2796. 10.1083/jcb.201904004 31296534 PMC6683751

[B9] Cherry P. D. Peach S. E. Hesselberth J. R. (2019). Multiple decay events target HAC1 mRNA during splicing to regulate the unfolded protein response. *Elife* 8:e42262 10.7554/eLife.42262 30874502 PMC6456296

[B10] Chow E. W. Clancey S. A. Billmyre R. B. Averette A. F. Granek J. A. Mieczkowski P. (2017). Elucidation of the calcineurin-Crz1 stress response transcriptional network in the human fungal pathogen *Cryptococcus neoformans*. *PLoS Genet.* 13:e1006667. 10.1371/journal.pgen.1006667 28376087 PMC5380312

[B11] Connolly S. Quasi-Woode D. Waldron L. Eberly C. Waters K. Muller E. M. (2018). Calcineurin regulatory subunit calcium-binding domains differentially contribute to calcineurin signaling in *Saccharomyces cerevisiae*. *Genetics* 209 801–813. 10.1534/genetics.118.300911 29735720 PMC6028253

[B12] Cyert M. S. Philpott C. C. (2013). Regulation of cation balance in *Saccharomyces cerevisiae*. *Genetics* 193 677–713. 10.1534/genetics.112.147207 23463800 PMC3583992

[B13] Dantas Ada S. Day A. Ikeh M. Kos I. Achan B. Quinn J. (2015). Oxidative stress responses in the human fungal pathogen, *Candida albicans*. *Biomolecules* 5 142–165. 10.3390/biom5010142 25723552 PMC4384116

[B14] de Castro P. A. Chiaratto J. Winkelströter L. K. Bom V. L. Ramalho L. N. Goldman M. H. (2014). The involvement of the Mid1/Cch1/Yvc1 calcium channels in *Aspergillus fumigatus* virulence. *PLoS One* 9:e103957. 10.1371/journal.pone.0103957 25083783 PMC4118995

[B15] Ghanegolmohammadi F. Yoshida M. Ohnuki S. Sukegawa Y. Okada H. Obara K. (2017). Systematic analysis of Ca(2+) homeostasis in *Saccharomyces cerevisiae* based on chemical-genetic interaction profiles. *Mol. Biol. Cell* 28 3415–3427. 10.1091/mbc.E17-04-0216 28566553 PMC5687040

[B16] Gomes-Neto J. C. Mantz S. Held K. Sinha R. Segura Munoz R. R. Schmaltz R. (2017). A real-time PCR assay for accurate quantification of the individual members of the altered schaedler flora microbiota in gnotobiotic mice. *J. Microbiol. Methods* 135 52–62. 10.1016/j.mimet.2017.02.003 28189782 PMC5365401

[B17] Granatiero V. Konrad C. Bredvik K. Manfredi G. Kawamata H. (2019). Nrf2 signaling links ER oxidative protein folding and calcium homeostasis in health and disease. *Life Sci. Alliance* 2 10.26508/lsa.201900563 31658977 PMC6819749

[B18] Iida K. Teng J. Cho T. Yoshikawa-Kimura S. Iida H. (2017). Post-translational processing and membrane translocation of the yeast regulatory Mid1 subunit of the Cch1/VGCC/NALCN cation channel family. *J. Biol. Chem.* 292 20570–20582. 10.1074/jbc.M117.810283 29042437 PMC5733593

[B19] Karababa M. Valentino E. Pardini G. Coste A. T. Bille J. Sanglard D. (2006). CRZ1, a target of the calcineurin pathway in *Candida albicans*. *Mol. Microbiol.* 59 1429–1451. 10.1111/j.1365-2958.2005.05037.x 16468987

[B20] Kumar V. Maity S. (2021). ER stress-sensor proteins and ER-mitochondrial crosstalk-signaling beyond (ER) stress response. *Biomolecules* 11:173. 10.3390/biom11020173 33525374 PMC7911976

[B21] LaFayette S. L. Collins C. Zaas A. K. Schell W. A. Betancourt-Quiroz M. Gunatilaka A. A. (2010). PKC signaling regulates drug resistance of the fungal pathogen *Candida albicans via* circuitry comprised of Mkc1, calcineurin, and Hsp90. *PLoS Pathog.* 6:e1001069. 10.1371/journal.ppat.1001069 20865172 PMC2928802

[B22] Li J. Yu Q. Zhang B. Xiao C. Ma T. Yi X. (2018). Stress-associated endoplasmic reticulum protein 1 (SERP1) and Atg8 synergistically regulate unfolded protein response (UPR) that is independent on autophagy in *Candida albicans*. *Int. J. Med. Microbiol.* 308 378–386. 10.1016/j.ijmm.2018.03.004 29544880

[B23] Li Y. Sun L. Lu C. Gong Y. Li M. Sun S. (2018). Promising antifungal targets against *Candida albicans* based on ion homeostasis. *Front. Cell. Infect. Microbiol.* 8:286. 10.3389/fcimb.2018.00286 30234023 PMC6131588

[B24] Liu S. Hou Y. Liu W. Lu C. Wang W. Sun S. (2015). Components of the calcium-calcineurin signaling pathway in fungal cells and their potential as antifungal targets. *Eukaryot Cell* 14 324–334. 10.1128/ec.00271-14 25636321 PMC4385803

[B25] Meng F. Yan J. Ma Q. Jiao Y. Han L. Xu J. (2018). Expression status and clinical significance of lncRNA APPAT in the progression of atherosclerosis. *PeerJ* 6:e4246. 10.7717/peerj.4246 29372117 PMC5775756

[B26] Mizuno T. Nakamura M. Irie K. (2018). Induction of Ptp2 and Cmp2 protein phosphatases is crucial for the adaptive response to ER stress in Saccharomyces cerevisiae. *Sci. Rep.* 8:13078. 10.1038/s41598-018-31413-6 30166606 PMC6117328

[B27] Mohsin A. A. Thompson J. Hu Y. Hollander J. Lesnefsky E. J. Chen Q. (2020). Endoplasmic reticulum stress-induced complex I defect: central role of calcium overload. *Arch. Biochem. Biophys.* 683:108299. 10.1016/j.abb.2020.108299 32061585 PMC7092715

[B28] Palmer C. P. Zhou X. L. Lin J. Loukin S. H. Kung C. Saimi Y. (2001). A TRP homolog in *Saccharomyces cerevisiae* forms an intracellular Ca(2+)-permeable channel in the yeast vacuolar membrane. *Proc. Natl. Acad. Sci. U.S.A.* 98 7801–7805. 10.1073/pnas.141036198 11427713 PMC35422

[B29] Park S. J. Kim Y. Yang S. M. Henderson M. J. Yang W. Lindahl M. (2019). Discovery of endoplasmic reticulum calcium stabilizers to rescue ER-stressed podocytes in nephrotic syndrome. *Proc. Natl. Acad. Sci. U.S.A.* 116 14154–14163. 10.1073/pnas.1813580116 31235574 PMC6628787

[B30] Peng L. Yu Q. Wei H. Zhu N. Ren T. Liang C. (2019). The TRP Ca(2+) channel Yvc1 regulates hyphal reactive oxygen species gradient for maintenance of polarized growth in *Candida albicans*. *Fungal Genet. Biol.* 133:103282. 10.1016/j.fgb.2019.103282 31629081

[B31] Peng L. Yu Q. Zhu H. Zhu N. Zhang B. Wei H. (2020). The V-ATPase regulates localization of the TRP Ca(2+) channel Yvc1 in response to oxidative stress in *Candida albicans*. *Int. J. Med. Microbiol.* 310:151466. 10.1016/j.ijmm.2020.151466 33291030

[B32] Priante G. Quaggio F. Gianesello L. Ceol M. Cristofaro R. Terrin L. (2018). Caspase-independent programmed cell death triggers Ca(2)PO(4) deposition in an *in vitro* model of nephrocalcinosis. *Biosci. Rep.* 38:BSR20171228. 10.1042/bsr20171228 29208768 PMC5770611

[B33] Rajakumar S. Bhanupriya N. Ravi C. Nachiappan V. (2016). Endoplasmic reticulum stress and calcium imbalance are involved in cadmium-induced lipid aberrancy in *Saccharomyces cerevisiae*. *Cell Stress Chaperones* 21 895–906. 10.1007/s12192-016-0714-4 27344570 PMC5003806

[B34] Rong F. Liu L. Zou C. Zeng J. Xu Y. (2020). MALAT1 promotes cell tumorigenicity through regulating miR-515-5p/EEF2 axis in non-small cell lung cancer. *Cancer Manag. Res.* 12 7691–7701. 10.2147/cmar.S242425 32943920 PMC7468487

[B35] Su X. Yan X. Chen X. Guo M. Xia Y. Cao Y. (2021). Calcofluor white hypersensitive proteins contribute to stress tolerance and pathogenicity in entomopathogenic fungus, *Metarhizium acridum*. *Pest Manag. Sci.* 77 1915–1924. 10.1002/ps.6218 33300230

[B36] Teng J. Iida K. Imai A. Nakano M. Tada T. Iida H. (2013). Hyperactive and hypoactive mutations in Cch1, a yeast homologue of the voltage-gated calcium-channel pore-forming subunit. *Microbiology (Reading)* 159(Pt 5) 970–979. 10.1099/mic.0.064030-0 23475949

[B37] Wu J. Chen Y. J. Dobbs N. Sakai T. Liou J. Miner J. J. (2019). STING-mediated disruption of calcium homeostasis chronically activates ER stress and primes T cell death. *J. Exp. Med.* 216 867–883. 10.1084/jem.20182192 30886058 PMC6446864

[B38] Xing H. Luo X. Li Y. Fan C. Liu N. Cui C. (2020). Effect of verapamil on the pharmacokinetics of hydroxycamptothecin and its potential mechanism. *Pharm. Biol.* 58 152–156. 10.1080/13880209.2020.1717550 31990625 PMC7034088

[B39] Xu H. Fang T. Omran R. P. Whiteway M. Jiang L. (2020). RNA sequencing reveals an additional Crz1-binding motif in promoters of its target genes in the human fungal pathogen *Candida albicans*. *Cell Commun. Signal.* 18:1. 10.1186/s12964-019-0473-9 31900175 PMC6942403

[B40] Xu H. Fang T. Yan H. Jiang L. (2019). The protein kinase Cmk2 negatively regulates the calcium/calcineurin signalling pathway and expression of calcium pump genes PMR1 and PMC1 in budding yeast. *Cell Commun. Signal.* 17:7. 10.1186/s12964-019-0320-z 30665402 PMC6341702

[B41] Yu Q. Ding X. Zhang B. Xu N. Jia C. Mao J. (2014a). Inhibitory effect of verapamil on *Candida albicans* hyphal development, adhesion and gastrointestinal colonization. *FEMS Yeast Res.* 14 633–641. 10.1111/1567-1364.12150 24650198

[B42] Yu Q. Zhang B. Yang B. Chen J. Wang H. Jia C. (2014b). Interaction among the vacuole, the mitochondria, and the oxidative stress response is governed by the transient receptor potential channel in *Candida albicans*. *Free Radic. Biol. Med.* 77 152–167. 10.1016/j.freeradbiomed.2014.09.011 25308698

[B43] Yu Q. Zhang B. Li J. Zhang B. Wang H. Li M. (2021). Corrigendum to “Endoplasmic reticulum-derived reactive oxygen species (ROS) is involved in toxicity of cell wall stress to *Candida albicans*” [Free Radic. Biol. Med. 99 (2016) 572-583]. *Free Radic. Biol. Med.* 162 176–178. 10.1016/j.freeradbiomed.2020.07.001 27650297

[B44] Zhang Z. Zhang L. Zhou L. Lei Y. Zhang Y. Huang C. (2019). Redox signaling and unfolded protein response coordinate cell fate decisions under ER stress. *Redox Biol.* 25:101047. 10.1016/j.redox.2018.11.005 30470534 PMC6859529

